# Benign idiopathic retroperitoneal cyst: A case series of three patients

**DOI:** 10.1016/j.ijscr.2019.07.050

**Published:** 2019-07-23

**Authors:** Danish Ali, Muhammad Zeeshan Sarwar, Fareeha Manzoor, Syed Asghar Naqi

**Affiliations:** a Lahore General Hospital/ PGMI, Lahore, Pakistan; b Mayo Hospital/King Edward Medical University Lahore, Pakistan

**Keywords:** Retroperitoneal cysts, Idiopathic benign cysts, Retroperitoneal mass, Case series/report

## Abstract

**Importance:**

Retroperitoneal cysts are a rare entity in the field of surgery and their management remains a clinical dilemma for many surgeons. This study addresses the clinical management of three benign retroperitoneal cysts which were presented in our hospital.

**Objectives:**

The objective of this case series is to explore the different clinical presentation of the retroperitoneal cyst (RPC). This study also aims at different tools of investigations especially role of CT scan in the diagnosis of RPC and finally the different treatment options available for RPC.

**Methods:**

This is a single centered case series of three retroperitoneal cysts which were presented in surgical department of our hospital in the year 2016 and 2017. Total three patients were presented in this case series, two of them were females and one was male. Females were 13 years and 23 years age-old, male was 45 years old. Two of them admitted through the Outdoor Patient Clinic and one was presented to the surgical emergency. Informed consent from the patients and guardian (case1) has been taken. Research work has been reported in line with the PROCESS criteria.

**Results:**

They were investigated and treated in our hospital. Two of them were diagnosed with retroperitoneal cysts preoperatively and one was diagnosed pre-operatively. In all three cases, surgery had been carried out and they had been observed in their early postoperative period and then followed up for two years. All biopsies had been followed and turned out to be benign idiopathic cysts. These patients have been followed up for 2 years after completion of their treatment.

**Conclusion:**

Retroperitoneal cysts in adults are an uncommon manifestation of abdominal pathology. Their presentation is quite vague. The rarity catches the surgeon off guard at times. Radiology plays a crucial role in defining these lesions and complete surgical excision remains the cornerstone of treatment for symptomatic and complicated patients.

## Highlights

• This case series is a good review of different variants of retroperitoneal cysts and their distinct morphological and clinical features, which would be helpful to differentiate among them.
• It emphasizes the symptomatology and different tools to investigate them preoperatively, especially the role of radiological investigations.
• It gives a detailed discussion of the different treatment options and their outcomes.
• This case series has been written in a very convenient but concise manner to make it easier for the readers to extract maximum out of it.

## 1 Introduction

Retroperitoneal cystic masses arise within the retroperitoneal space, but it does not share an origin with any solid organ [1]. They are an infrequent occurrence in surgical practice with an incidence in available literature of 1/5750–1/250,0002. The retroperitoneum is a potentially vast space and hence, masses forming in it can grow to substantially large sizes before they present clinically. Their symptoms are vague and are mainly due to pressure on adjacent structures. CT is ideal for the assessment of retroperitoneal disease because it provides discrete sectional images of the organs and retroperitoneal compartments [1]. Surgical resection remains the mainstay of treatment. We present here a series of three cases mimicking various intra-abdominal pathologies but turned out to be benign cysts in the retroperitoneum.

## 2 Case series

### 2.1 Case 1

**Fig. 1:**
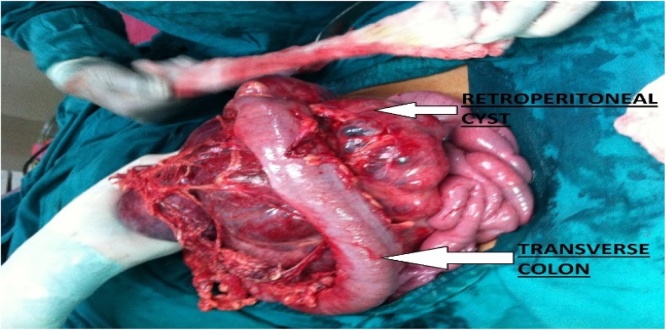
Per op findings of cyst (case 1).

**Fig. 2:**
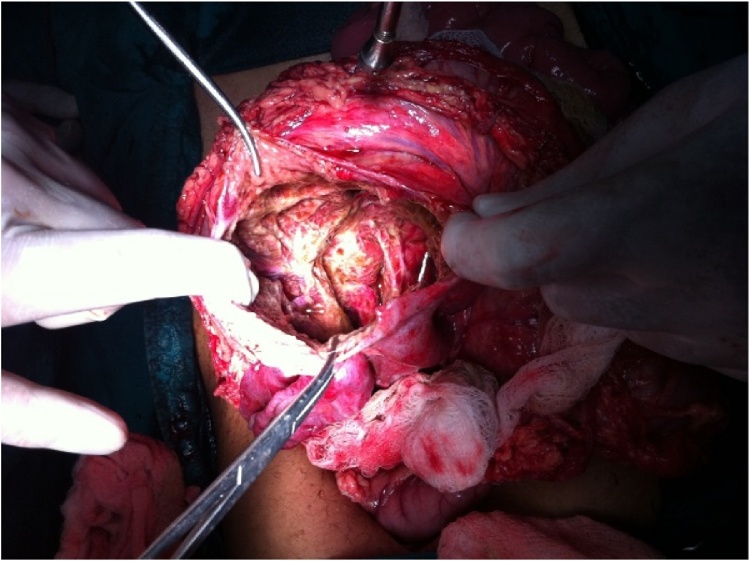
Opening the wall of cyst (case 1).

A 13-year-old young female presented to the emergency department with abdominal pain, constipation and repeated bouts of vomiting for the last three days. The pain was sudden in onset, colicky in character and intermittent in onset. Her past medical history showed that she was treated with anti-tuberculous therapy for pulmonary tuberculosis. She took anti-tuberculosis treatment for nine months and completed this course two years back. General physical examination showed a heart rate of 110 beats/min and a BP of 90/60 mmHg with obvious clinical signs of dehydration. Abdominal examination revealed mild distension and tenderness with a 5 × 10 cm intra-abdominal mass in the upper abdomen. Bowel sounds were exaggerated. Laboratory results showed a hemoglobin level of 8.7 g/dl and a Serum Creatinine of 1.2 mg/dl. X-ray abdomen showed air-fluid levels in the small bowel. Ultrasonography of the abdomen revealed a 15 × 18 cm hypoechoic mass in the center of the abdomen. These findings were suggestive of small bowel obstruction secondary to abdominal tuberculosis. The patient was resuscitated, and surgical exploration was planned. Exploration revealed a huge cyst with dimensions of 24 cm × 20 cm × 16 cm, arising from the retroperitoneum, projects into transverse mesocolon and displacing loops of the small intestine (Fig. 1, Fig. 2). Cyst contained three liters of serous fluid. Complete excision was possible without resection of the associated organs. The specimen was sent for histopathology. Inspection of the specimen revealed 24 × 12 × 10 cm cyst, with a smooth outer surface and an irregular inner surface. Microscopic examination confirmed the diagnosis of the benign retroperitoneal cyst was made. There was no atypia or malignancy. She was discharged on her 3rd postoperative day. Post-op recovery was uneventful. The patient remains asymptomatic during the two years follow up period.

### 2.2 Case 2

**Fig. 3:**
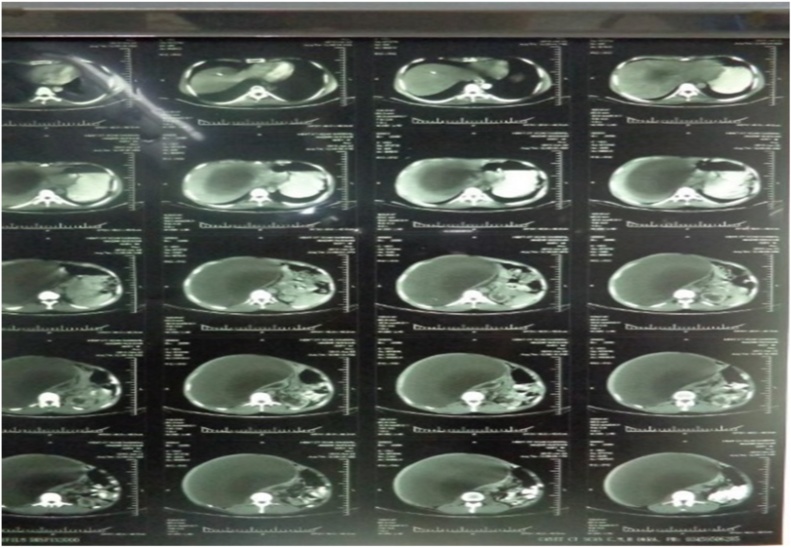
CT scan showing retroperitoneal Cyst displacing right kidney (case 2).

A 45-year-old male presented to the outpatient clinic with abdominal distension and a central abdominal mass of three years duration. Mass is progressively increasing in size without any symptoms. His past medical history was non-contributory. General physical examination was unremarkable. Abdominal examination revealed a large, non-tender mass in the central abdomen which was extending to the right hypochondrium. All laboratory tests were normal. CT scan showed a huge retroperitoneal cyst occupying right hemiabdomen. The cyst was well circumscribed, unilocular and containing homogenous fluid in it. It was displacing right kidney (Fig. 3). A provisional diagnosis of the retroperitoneal cyst was made, and surgical excision was planned. On exploration, there was a large retroperitoneal cyst measuring 18 × 12 × 15 cm in size, displacing right kidney towards the midline and loops of the small intestine to the left. Fascial planes were well preserved. Complete excision of the cyst was achieved. The specimen was sent for histopathology. Inspection of the specimen revealed 16 × 10 × 09 cm cyst, with a smooth outer surface and an irregular inner surface. Microscopic, Diagnosis of the benign retroperitoneal cyst was made. There was no atypia or malignancy seen. He was discharged on 4th postoperative day. Post-op recovery was uneventful. The patient remains asymptomatic during the two years follow up period.

### 2.3 Case 3

**Fig. 4:**
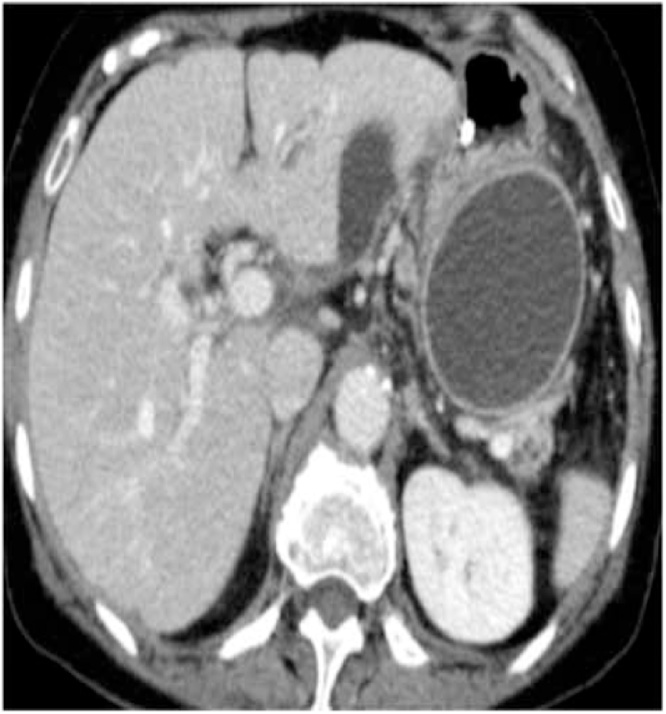
CT scan showing cyst In left hypochondrium (case 3).

**Fig. 5:**
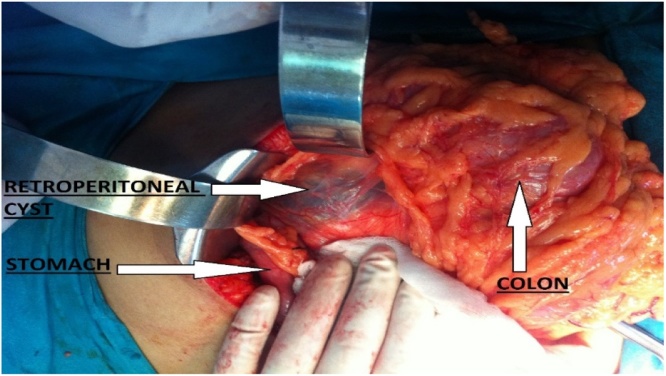
Per op findings of the cyst (case 3).

A 23-year-old female presented to the outpatient clinic with heaviness and epigastric discomfort of 2 years duration. She was under ultrasound surveillance in some other hospital. She denied any history of upper abdominal pain suggestive of acute pancreatitis. She underwent ultrasound-guided aspiration three times revealing two liters of clear fluid on each occasion. No record of biochemistry and cytology was available. All laboratory tests were normal including serum amylase level. On abdominal examination, there was fullness in the left hypochondrium. CT scan abdomen and pelvis showed the presence of a retroperitoneal cyst in the left hypochondrium, 15 × 10 × 12 cm in size with no mass effect on surrounding structures (Fig. 4). It was not originating from any sold organ or structure. Diagnosis of the retroperitoneal cyst was made. Exploratory laparotomy was performed via left subcostal incision and complete excision was carried out successfully (Fig. 5). Inspection of the specimen revealed 14 × 10 × 10 cm cyst, with a smooth outer surface and an irregular inner surface. On microscopic examination, Diagnosis of the benign retroperitoneal cyst was made. There was no atypia or malignancy seen. She was discharged on her 3rdpostoperative day. Post-op recovery was uneventful. The patient remains asymptomatic during the two years follow up period.

## 3 Discussion

The embryological and histopathological basis allows retroperitoneal cysts to be divided into the following categories: (1) Urogenital; (2) Meso-colic; (3) Cysts arising in cell inclusions; (4) Traumatic; (5) Parasitic; (6) Lymphatic [3,4]. This classification is based on the premise that the cysts described are present in the retroperitoneal space and are only linked to an adult anatomical structure by means of intervening loose areolar tissue [5]. Cysts in our cases were benign idiopathic and they did not fall in the above-mentioned categories.

Its diagnosis remains elusive in terms of clinical symptomatology and poses a great challenge to the unsuspecting physician. About 50% of the cases usually present with vague symptoms like abdominal pain and distension [5] while one-third of the presentations are of an incidental nature [5,7]. Other non-specific symptoms can include back pain, weight loss, lower limb swelling and referred pain to the lower limbs due to pressure on adjoining structures. In this case series, patients present with vague abdominal pain, abdominal mass, and epigastric fullness. These symptoms were mainly due to pressure into adjacent structures.

Radiology serves as an indispensable tool to delineate these lesions and investigations including Computed Tomography are often required to define these lesions appropriately [1]. It provides discrete sectional images of the organs and retroperitoneal compartments. It gives valuable information about cyst size, location, and shape. CT can help to find the presence and thickness of the wall, septa, calcification, and fat. It may also show the involvement of adjacent organs. Familiarity with the most relevant radiologic features, in combination with clinical information, allows adequate lesion characterization [1]. In our cases, CT was able to provide information about cysts sizes, shape and their site of origin and involvement of adjacent structures. Other imaging modality like Magnetic Resonance Imaging may also be useful in preoperative localization and differentiation of the retroperitoneal cyst [8].

Surgical intervention is required in cases where these cysts tend to become symptomatic and when inherent complications like perforation, infection, and malignancy are expected. Both trans-abdominal and extra-peritoneal approaches can be used to approach and excise the cyst depending on the location [6]. A laparoscopic approach can also be undertaken in selected cases if enough expertise is available [9]. We use a trans-abdominal approach in our cases because we were not sure about the diagnosis (case 1) and we feel comfortable (case 2, 3).

The treatment of choice, however, remains complete excision with preservation of surrounding important structures it is related to [2]. Other less acceptable options include marsupialization, fenestration and partial excision when complete excision is not possible, or the cyst is infected [10]. In such cases recurrence is a cause of major concern. Care must be taken during dissection as dissemination in the retroperitoneal space is rare, but a likely fatal complication [11].

## 4 Conclusion

Retroperitoneal cysts in adults are an uncommon manifestation of abdominal pathology. The presentation is varied between asymptomatic to vague abdominal symptoms. The rarity catches the surgeon off guard at times. Radiology plays a crucial role in defining these lesions and complete surgical excision remains the cornerstone of treatment for symptomatic and complicated patients.

## 5 Limitations

This study had a small number of cases. It was not a randomized clinical trial. This study was done in a single center.

**Note**: Research work has been reported in line with the PROCESS criteria [12].

## Sources of funding

No source of funding.

## Ethical approval

Study was discussed in institutional review board of King Edward Medical University, Lahore and got approval from board with reference No.154 / RC/ KEMU Date: 01/ 01/2016.

## Consent

Informed consent from the patients, guardian (case 1) and institutional board has been taken.

## Author’s contribution

**Danish Ali**: Conceptualization, Methodology, Writing the original paper, Project administration

**Muhammad Zeeshan Sarwar**: Study concept, Review & editing the draft, Project administration, and Supervision.

**Fareeeha Manzoor**: Data collection, Methadology, Data interpretation.

**Syed Asghar Naqi**: Supervision.

## Registration of research studies

https://www.researchregistry.com/browse-the-registry.

Research Registry UIN researchregistry4716.

## Guarantor

Danish Ali.

Muhammad Zeeshan Sarwar.

## Provenance and peer review

Not commissioned.

## Declaration of Competing Interest

None.
